# Indirect Estimates of Total Fertility Rate Using Child Woman/Ratio: A Comparison with the Bogue-Palmore Method

**DOI:** 10.1371/journal.pone.0067226

**Published:** 2013-06-24

**Authors:** Matt Hauer, Jack Baker, Warren Brown

**Affiliations:** 1 Carl Vinson Institute of Government, University of Georgia, Athens, Georgia, United States of America; 2 UNM Geospatial and Population Studies, University of New Mexico, Albuquerque, New Mexico, United States of America; 3 Cornell Institute for Social and Economic Research, Cornell University, Ithaca, New York, United States of America; IPATIMUP (Institute of Molecular Pathology and Immunology of the University of Porto), Portugal

## Abstract

Indirect estimation methodologies of the total fertility rate (TFR) have a long history within demography and have provided important techniques applied demographers can use when data is sparse or lacking. However new methodologies for approximating the total fertility rate have not been proposed in nearly 30 years. This study presents a novel method for indirectly approximating the total fertility rate using an algebraic rearrangement of the general fertility rate (GFR) through the known relationship between GFR and TFR. It then compares the proposed method to the well-known Bogue-Palmore method. These methods are compared in 196 countries and include overall errors as well as characteristics of the countries that contribute to fertility behavior. Additionally, these methods were compared geographically to find any geographical patterns. We find this novel method is not only simpler than the Bogue-Palmore method, requiring fewer data inputs, but also has reduced algebraic and absolute errors when compared with the Bogue-Palmore method and specifically outperforms the Bogue-Palmore method in developing countries. We find that our novel method may be useful estimation procedure for demographers.

## Introduction

Estimates of population fertility characteristics are of critical importance for understanding short-term shifts in population age-structure and related growth dynamics [1]–[3]. They are utilized in creating important demographic summary indicators such as total fertility rate (TFR), Euler-Lotka growth parameters, reproductive value, and net-reproductive rates [1]–[6]. In spite of their obvious importance, enduring challenges associated with the collection of vital data records have often left demographers with a need to estimate these patterns using incomplete data [7]–[9]. Methods of *indirect estimation*–based on either augmentation of incomplete data or reliance upon decomposition or algebraic reformulation of known demographic relationships to measure population processes–have a long history in demography [7]–[14]. The under-reporting of births, in particular, has generated a significant amount of research reporting methods for adjusting age-specific fertility rates in light of survey data [9], [10]–[13], the application of stable population models and associated model fertility schedules [6]–[7], [14], or the utilization of regression-based methods in conjunction with censal age-structures or indirect indicators of fertility variation such as marital status [15]–[19]. In spite of the large number of proposed approaches, relatively little is known about how accurate such estimates are in general, or how their performance might vary between simple and more complex methods [20]–[21]. This paper attempts to fill this gap in the demographic literature by comparing the performance of a regression-based method for estimating total fertility rate (TFR) based on symptomatic indicators [15]–[16] to a simpler method requiring only censal age-structures and algebraic rearrangement to convert the child/woman ratio into an estimate of TFR.

Regression-based methods, such as the Bogue-Palmore procedure, rely upon the powerful least-squares criteria to predict TFR in light of symptomatic indicators [15]–[18], [22]–[23]. Relatively little data is required, making the method an attractive procedure for estimating TFR in contexts where vital records data collection is known to be problematic or incomplete; specifically, these methods have relied upon relatively few data inputs such as the child/woman ratio and event-history (life-table) values [18], [22]. While more well-known fertility estimation methods can often require substantial modeling effort and a clear understanding of how survey data are formulated [6]–[14], regression-based methods often require only the application of a set of coefficients to indicator variables to make TFR estimates [15]–[16], [23]. Estimates of TFR using indirect indicators and a regression approach have been characterized by mixed results: often it is difficult to obtain even accurate data on the indirect indicators [12], [16], [23] and the application of regression coefficients formulated at large geographic scales to estimating fertility at smaller geographic scales–which may also be more rapidly changing over time [18]–has been problematic [23]–[24]. These challenges suggest that simpler approaches might be more fruitful when estimating TFR.

In spite of known relationships between child/woman ratios and gross reproductive rate [3], [15]–[18], [23] and the straightforward relationship between GRR and TFR [3], [23], to date no study has explored the use of simple estimators that leverage this relationship through algebraic rearrangement aimed at transforming child/woman ratios into estimates of TFR. While such a method would be subject to under-reporting of small children within Censuses [22]–[23], it would not require any specific data on vital rates from surveys or other forms of demographic surveillance, nor would it require data such as marital status, life-expectancy, or infant mortality rates. As a stand-alone method, if a simple algebraic rearrangement could produce at least equally accurate estimates to those resulting from more data-intensive procedures then it would have much to recommend it, especially in developing countries plagued by under-surveillance of births [6]–[9], [10]–[13] and a lack of collection of data on appropriate symptomatic indicators. It would also address the shortcomings associated with applying national-level regression coefficients to subnational geographic levels [22]–[24] and permit local-level tailoring of TFR estimates based on the moments of a female age-distribution that is allowed to vary locally along with the total fertility rates that it predicts. This paper develops such an algebraic method and then evaluates it at the National level using data from 184 countries, drawn from the most recent UN Population data (http://esa.un.org/unpd/wpp/Excel-Data/fertility.htm). The performance of the method is characterized and then compared (where data permit) to that of the Bogue-Palmore method. Statistical measures of accuracy and bias such as the mean absolute and algebraic percentage errors (MAPE & MALPE) [21], [25]–[26] and the root mean-squared error (RMSE) [27]–[28] are computed, comparing the estimated values to those reported in the IDB. The relative performance of each method is considered in light of the characteristics of countries where each method is more accurate, by considering data from the Demographic and Health Surveys (DHS–www.measuredhs.com) on population characteristics. The results are considered in light of their implication for the formulation of demographic estimates with incomplete data and through map-based presentation of variation in accuracy.

## Materials and Methods

### Demographic Relationships: Child/Woman Ratio to Total Fertility Rate

The method described here relies upon known relationships between TFR and the General Fertility Rate [3], [23]. TFR is calculated as the sum of age-specific fertilities multiplied against the width of age interval of interest, typically a five-year grouping:
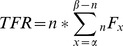
(1)


Where 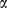 and 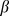 are the minimum and maximum ages at childbearing.

_n_F_x_ is equal to the ratio of births to the population at risk for giving birth:
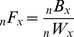
(2)



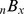
 = total number of Births to women aged x to x +n.


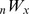
 = total number of women aged x to x+n.

The relationship of TFR to the General Fertility Rate (GFR) is well known. The GFR is calculated as the ratio of births observed among women of childbearing ages:
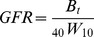
(3)


Where B_t_ is the total number of births and _40_W_10_ is the number of women aged 10 to 50. We can rewrite the GFR equation to be in the same notation as _n_F_x_,
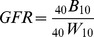
(4)


Which restates the GFR in a form where one may insert the GFR function into the TFR function and just multiply by the width of the interval to approximate the Total Fertility Rate. Summing across age intervals, as is the case with TFR, is unnecessary since the age intervals are simplified into one single age interval. Mathematically, the sum of the ratios (TFR) is different from the ratio of the sums (GFR), but empirically the results should be and are very similar [3].
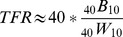
(5)


Age-specific Fertility Rates are commonly calculated in one or five year intervals, denoted as 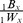 and 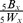. Five-year ASFRs can thus be rethought of as the sum of births for women aged x to x+n divided by the sum of women aged x to x+n. The age-specific fertility rate for women aged 20 to 24 could thus be written as
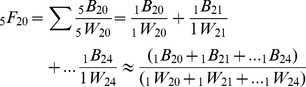
(6)


Table 1 utilizes data from the Human Fertility Database [29] for Austria for the year 2010. We can see how _n_F_x_ for a five-year interval is related to the sum of its individual age groups and thus the age-specific fertility rate can be approximated as both the sum of the ratios and as the ratio of the sums. Equation 5 simply extrapolates the TFR as the ratio of the sums for the entire life of reproduction, menarche to menopause to create an approximation of the TFR.

**Table 1:** ASFRs for Austria, 2010.

Age	Population	Births	ASFR
**20**	50,505	1,601	0.032
**21**	51,325	2,073	0.04
**22**	51,796	2,480	0.048
**23**	51,803	2,916	0.056
**24**	52,582	3,391	0.064
**20–24**	258,010	12,461	0.241
**Sum of ASFRs**			0.241

It should be noted that Bogue and Palmore [15] observed the strong relationship between GFR and TFR when they published their first indirect estimation method in 1964. They found the correlation between GFR and TFR to have an r^2^ = 0.992 *at the national level*. This was later confirmed by Tuchfield et al [24] a decade later when they found GFR and TFR to have r^2^ = 0.943 for 1970 *at the US county level*.

With certain strong assumptions including no infant mortality and no migration in the previous five-years, the child/woman ratio may be substituted into the term representing the General Fertility in equation 5 and used to estimate TFR as:
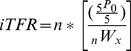
(7)


Where 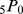 is equal to the Population aged 0 to 4.

This derivation we have dubbed the implied Total Fertility Rate (iTFR) due to it reflecting the implied total fertility rate present in the age structure of the population. It suggests that, subject to the aforementioned assumptions, the child/woman ratio may be used directly to make an approximation of TFR. The iTFR is nothing more than an extension of the algebra already used to construct grouped rates extrapolated out to include the entire fertility interval. Instead of 40 1-year ASFRs, or 8 5-year ASFRs, or 4 10-year ASFRs, we are simply calculating 1 40-year ASFR. And since it accommodates the entire fertility interval (menarche to menopause), 1 40-year ASFR essentially is the TFR, simply constructed as a single interval instead of multiple intervals with the assumption that ASFRs are constant over time, keeping in the tradition of Brass’s P/F ratio [30], Arriaga’s variant adjustments for temporal trends in ASFR [31], and other indirect fertility estimation techniques namely Bogue and Palmore [15], Palmore [16], and Rele [17].

The Hamilton-Perry method [32] for population projections utilizes a similar approach for projecting the populations aged 0–4 and 5–9. Hamilton-Perry projects these populations utilizing the Child-Woman Ratio multiplied by the previously projected women of child-bearing ages.
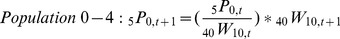
(8)Where


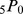
 is the Population aged 0 to 4.


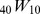
 is the female population aged 10 to 50.

The Hamilton-Perry method essentially projects child populations through the use of a fertility wide (menarche to menopause) ratio of children to women. Equation 7 utilizes the established relationship between CWR/GFR and TFR to directly convert the Child-Woman Ratio into a Total Fertility Rate.

Given the strong observed relationship between GFR and TFR [15], [24], given that Hamilton-Perry [32] utilizes the same relationship to project populations, and given that the strong assumption that fertility rates remain constant over time, as supported through the literature [15], [16], [17], [30], [31], we will now compare the Bogue-Palmore method with equation 7, the implied Total Fertility Rate, to determine the accuracy of the method proposed here.

### Database and Demographic Estimates

Data on populations at the country level, including population counts used to estimate the child/woman ratio, levels of infant mortality, the proportion of women married, and estimates of TFR were taken from the United Nations Population Division for the year 2000 [33]. It should be recognized in considering these inputs that many of the country-level inputs are based on demographic remediation methods as well [8]. In light of this, it may seem best to consider the country-level estimates made here as measuring the ability of each method to replicate the modeled estimates of TFR provided by the UN, rather than a gold-standard estimate of such. The child/woman ratio based estimator described above was used to estimate TFR for 184 countries worldwide, it was compared to the regression-based approach of Bogue and Palmore [15] as revised in 1978 by Palmore [16]. The Bogue-Palmore approach utilizes regression to predict GFR based on its relationship with the child/woman ratio (based on the same logic outlined in our algebraic approach) as well as on the proportion of women married in the last five years and the infant mortality rate. The estimating function is based on national-level estimation of coefficients 15–16 applied to each nation in this study based on the regression formulation:
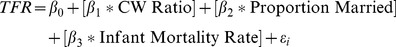
(9)


The method provides estimates of B_0_, B_1_, etc. that were fitted using standard least-squares criteria [34]–[35] at the national level as reported in Palmore [16]. The estimated regression coefficients of Palmore [16] were utilized here to make estimates for 150 of the 196 countries considered in this analysis. While characterization of errors associated with each method were made for all countries for which estimates could be made, comparisons of relative performance between the child/woman ratio method and the Bogue-Palmore estimates were restricted to those countries for which data were available to make Bogue-Palmore estimates (n = 54).

### Evaluation Statistics

In evaluating the accuracy of population estimates, applied demographers typically apply *ex-post-facto* evaluation statistics–those which compare estimated values to those of the target Census year [21], [26], based on numeric or percentage discrepancies. In this study, comparisons of absolute and signed numeric and percentage differences, and estimates of moments of these distributions such as averages or the root mean-squared error [27]–[28] were employed to evaluate the accuracy and bias associated with estimates of TFR in these estimates. Signed, algebraic errors (mean algebraic error–MAlgE–and mean algebraic percentage error–MALPE) proposed to capture bias [21], [25] while absolute numeric (mean absolute error–MAE) and percentage (MAPE) were utilized to capture accuracy. The Root Mean Squared Error (RMSE) is reported in both numeric and percentage terms as a measure of the robustness of the method across the diverse nations for which it was tested [27]–[28]; a robust estimate should have a relatively low RMSE indicating the presence of few unusually large errors. Here, we summarize results in percentage terms to avoid size-related bias in numeric comparisons [21], [25], [36]. In this analysis, comparisons of the population characteristics –suggested to be associated with fertility variation in the Proximate Determinants model of Bongaarts and Potter [19]– associated with countries where each method (the algebraic percentage error scores in particular) performed best were made using contingency table analysis based on the chi-squared statistic [37]–[38] and the Wilcoxon ranked sums test [39].

## Results

**Figure 1:**
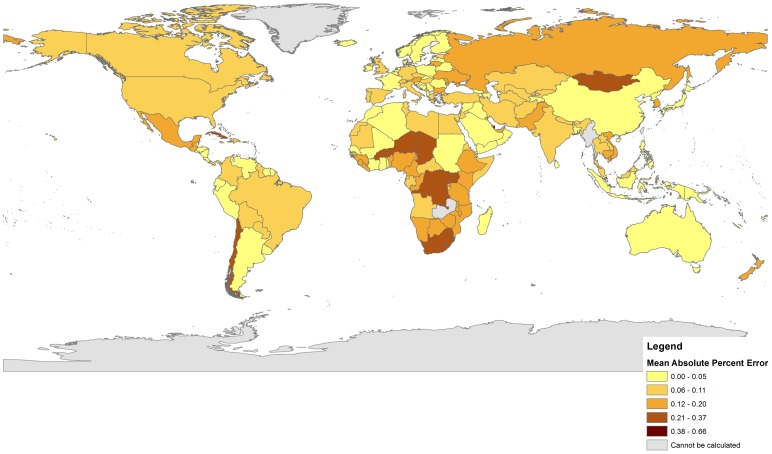
Absolute Percent Errors for Bogue-Palmore Method. The darker the country, the larger the absolute percent error. Countries in gray could not be calculated due to data limitations.

**Figure 2:**
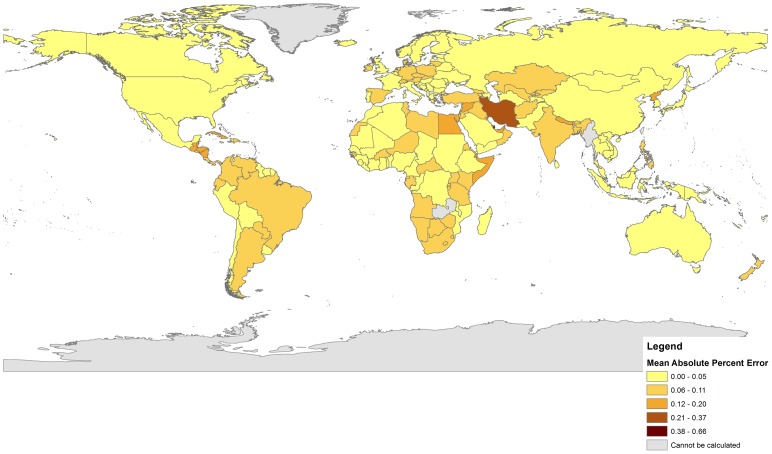
Absolute Percent Errors for the Child Woman Ratio. The darker the country, the larger the absolute percent error. Countries in gray could not be calculated due to data limitations.

Table 2 reports the overall performance of each method across 196 countries included in this analysis. Table 3 compares the population characteristics of the countries in which each method performed best. Figures 1 and 2 provide graphical reviews in chloropleth maps of the mean absolute percentage error (MAPE) associated with each set of estimates (Figure 1–Bogue-Palmore and Figure 2–Child/Woman Ratio) at the national level. Overall (Table 2), the Child/Woman Ratio method presented a lower average error (MAPE = 5.71%) than the Bogue-Palmore estimates (MAPE = 9.60%). This suggests an average percentage point improvement of 3.89 points when using the child/woman ratio procedure. While both methods tended to underestimate the UN values for TFR, the Bogue-Palmore method appeared to be less biased on average (MALPE = 0.92%) than the child/woman ratio (MALPE = 1.89%). The child/woman ratio method, however, appears to be much more robust than the Bogue-Palmore procedure: the RMSE associated with the former is only 7.35%, compared to 27.11% for the latter. This represents a nearly four-fold improvement in accuracy when using the child/woman ratio method.

**Table 2:** Performance by Method: Child Woman Ratio and Bogue-Palmore Methods.

	Child Woman Ratio Method				Bogue-Palmore Method		
n	MAPE	MALPE	RMSE	Percentage PointImprovement (Average)	MAPE	MALPE	RMSE
196	5.71%	1.89%	7.35%	3.89	9.60%	0.92%	27.11%

**Table 3:** Population Characteristics Associated with Greater Performance: Child Woman Ratio vs. Bogue-Palmore Methods.

		Number of Countries With Highest Performance		Average of DHS Estimates		
n	Population Characteristic	CW	BP	CW	BP	p-value of t-test
150	Total Fertility Rate	95	55	2.87	2.41	**0.023**
						**Z-value of Wilcoxon Test**
43	House has Electricity	29	14	40.59%	72.86%	**0.005**
39	Percent Literate	26	13	57.35%	75.82%	0.081
48	Percent Births in Health Facility (3 years prior)	28	20	56.33%	63.39%	0.358
35	Percent of Women Using Contraceptives	23	12	49.50%	63.29%	*0.063*
41	Percent of Women Never Married	26	15	27.37%	29.31%	0.199
47	Infant Mortality Rate Previous 5 Years	28	19	35/10,000	20/10,000	**0.005**
145	Infant Mortality Rate (CIA Factbook)	95	55	28/10,000	29/10,000	0.3069

The performance of each method appears to depend on a number of the proximate determinants of fertility (Table 3). Comparisons are limited by the inability to compute TFR using the Bogue-Palmore method in a significant number of countries as well as by availability in the Demographic and Health Surveys datasets. However, where comparisons can be made, using a simple t-test of means for the Total Fertility Rate and the Wilcoxon signed rank sum test for the other variables, it appears that the Child/Woman Ratio method out-performs the Bogue-Palmore method in countries where: TFR is higher (p = 0.023), the proportion of houses with electricity is lower (p = 0.005), and infant mortality rates are higher (p = 0.005 using DHS data). Differences in the percentage of births occurring in a health facility had no impact on performance (p = 0.358), nor did the percent of women never married (p = 0.199). It is noteworthy that the percentage of women using contraceptives appears to be lower in areas where the Child/Woman Ratio method out-performs, though the p-value is at the threshold of the strict alpha = 0.05 significance level (p = 0.063) as well as the percentage of the population that is literate (p = 0.081). Infant mortality had an interesting impact on performance with DHS data showing that the Child/Woman Ratio method out-performing the Bogue-Palmore method in areas with higher infant mortality (p = 0.006) but was insignificant while using the CIA World Factbook data (p = 0.3069). Despite these differences in the impact of infant mortality rate on the estimates, the Child/Woman Ratio method still outperformed the Bogue-Palmore method in the majority of countries for both data sources (28 to 19 using DHS data and 95 to 55 with CIA World Factbook data).

Figures 1 and 2 provide interesting perspectives on the geography of performance difference that is best considered in light of the analysis provided in Table 3. Figure 1 (mapping the performance of the Bogue-Palmore method) suggests significant spatial heterogeneity; the method tends to perform least well in sub-Saharan Africa as well as in Russia, Mongolia, and parts of Eastern Europe and South-Asia (darker colors indicate larger percentage errors). In contrast, Figure 2 suggests that the Child/Woman Ratio method is characterized by much less significant spatial clustering of performance. While Iran presents a notable exception in terms of performance, the low amplitude of color variation suggests that the method performs equally well across a wide variety of settings. It is worth recalling that the RMSE score for the Child/Woman Ratio method is considerably lower (7.89%) than that associated with the Bogue-Palmore method (27.11%). Figure 1 suggests that much of the higher variability may come from the darker-colored regions in sub-Saharan Africa, Eastern Europe and parts of Asia. Table 3 suggests that the map-based differences may relate to rather obvious differences in standard of living and associated governmental surveillance of vital rates.

## Discussion

The results of this study suggest that when compared to the Bogue-Palmore method, dramatic improvements in the accuracy of estimates of TFR may be made by employing a simple method based only on the child/woman ratio. The method assumes no migration among women of child-bearing age and their dependent young aged 0 to 4 years, and insignificant infant mortality. These results are surprising given the strong assumptions present in the method and may speak to the value of locally-tailored demographic estimates instead of estimates that minimize error over large, diverse, settings that may have little in common with one another. A lack of consideration of locally-specific error measures in standard least-squares estimation algorithms [40]–[41] may be an important contributor to these results as regression-based methods such as the Bogue-Palmore depend explicitly on this criteria in a global sense. Alternatively, it is possible that the regression coefficients estimated by Bogue and Palmore [15]–[16] may be out of date–in other words, relationships between child/woman ratio, marriage status, and infant mortality may not be the same today as they were when first estimated in these papers. Given the striking observed shifts in demographic patterns over the last 40–50 years [42] this seems to be a plausible explanation for why the Child/Woman ratio based method might out-perform. A reanalysis of these relationships in current times might yield different results from those reported in this study and would be a fruitful further avenue of research in demography.

In spite of these encouraging results, it is worth noting that the use of child/woman ratio for approximating TFR is subject to some important limitations that demographers should consider when implementing the method. It is obvious that successful use of the method is dependent upon accurate census enumeration data. Unfortunately, it is known that enumerations of children aged 0–4 years are biased by age-heaping [23] and although this effect may be remediated through smoothing techniques [8]–[9], [23] the well-known, but poorly-understood phenomenon of maternal under-reporting of small children [23]is much more difficult to address. As Hanenberg [23] points out, the undercounting of whole families may not affect measurement of the child/woman ratio as long as families are missing at random. However, presently little data exists to evaluate whether census omissions in developing nations will produce data that is “missing at random”, or which is systematically dependent on specific characteristics. Previously, Hanenberg [23] and others [7] have expressed suspicion about the assumption that missed enumerations are missing at random and not systematically dependent upon population characteristics. The authors of this paper share that suspicion and suggest careful consideration of the quality of census enumerations when utilizing the Child/Woman Ratio method.

Furthermore, the evaluation made in this paper depended on two forms of uncertain or omitted data in its comparisons. First, symptomatic indicator data were not available for all countries, limiting the ability to compute Bogue-Palmore estimates in all nations for which TFR estimates were available. This would pose (at least conceptual) limitations on the results presented here in two ways. First, the estimation of average errors associated with the Bogue-Palmore estimates could be directionally-biased by this lack of availability if this availability and the potential performance of the method were systematically related. In this sense, the performance of the Bogue-Palmore estimates could be either better or worse if data were available to make these estimates. In other words, the performance observed here is a function of both the appropriateness of the original regression model for the countries analyzed as well as the limitations of the sample of countries for which the method could be used. The greater availability of the simple data used in the Child/Woman ratio method speaks to its practical utility and certain advantages over the Bogue-Palmore method; however, the omission of n = 46 countries from the analysis is not to be underappreciated. While the average errors associated with the Child/Woman ratio method is likely robust, the errors associated with the Bogue-Palmore method cannot be certainly characterized as such. Figure 1 reveals graphically that many of the omissions are in Africa, Eastern Europe, and Asia: the geographic regions where the method appeared to perform most poorly. While not definitive, this does suggest that if these countries could be included it might serve only to degrade the overall performance of the method. In this case, it might be true that the Child/Woman ratio method *is even better in a relative performance sense than the results presented here suggest.*

In addition to these limitations, it should also be noted that both methods were used to predict a TFR that was itself estimated by the United Nations. It is largely agreed within the demographic community that the UN estimates are likely the best available estimates, but it should be remembered that these estimates themselves are subject to remediations for incomplete data [8], [38]. As such, the comparisons made here should be likened to asking the question of which method most accurately recreates the best available estimates of TFR at present. This is a common problem in indirect estimation [7]–[9], where gold-standards for comparison are not always available. In the case of TFR, comparisons to the number of births predicted by the estimate (one potential way to compare fertility estimates to Census data) would only be made possible with further, uncertain decompositions of the TFR into age-specific fertility schedules. For now, it appears that the results of study such as the one reported here are the only mechanism for evaluating the Child/Woman Ratio method, but it should be remembered that in the context of this study “accuracy” and “consistency with best-available estimates” are synonymous.

In spite of these limitations, the successful application of a very simple method of approximating TFR using the child/woman ratio in conjunction with simple algebraic rearrangement is highly promising. If one may recreate the UN-based estimates using such simple procedures it suggests that applied demographers working with limited resources may make very successful use of this simple technique in creating quality estimates of an important demographic indicator that is used to summarize fertility patterns in a meaningful way, similar to the way that life-expectancy provides summarized information on mortality. The method may also be locally-tailored to specific circumstances where vital-records data are not collected at all and such local-tailoring may even suggest the successful extension of the method to sub-national levels where the Bogue-Palmore approach has been problematic [23], [24]. When considering its simplicity and low data requirements, it appears that the Child/Woman ratio method presented in this paper may be a useful estimation procedure worthy of further consideration in demography.
